# Time-Restricted Feeding Improves Circadian Dysfunction as well as Motor Symptoms in the Q175 Mouse Model of Huntington’s Disease

**DOI:** 10.1523/ENEURO.0431-17.2017

**Published:** 2018-01-03

**Authors:** Huei-Bin Wang, Dawn H. Loh, Daniel S. Whittaker, Tamara Cutler, David Howland, Christopher S. Colwell

**Affiliations:** 1 Department of Psychiatry and Biobehavioral Sciences, University of California - Los Angeles, Los Angeles, CA 90024-1759; 2 CHDI Foundation, Princeton, NJ 08540

**Keywords:** time-restricted feeding, fast/feed cycle, circadian rhythms, Huntington’s disease, Q175

## Abstract

Huntington’s disease (HD) patients suffer from a progressive neurodegeneration that results in cognitive, psychiatric, cardiovascular, and motor dysfunction. Disturbances in sleep/wake cycles are common among HD patients with reports of delayed sleep onset, frequent bedtime awakenings, and fatigue during the day. The heterozygous Q175 mouse model of HD has been shown to phenocopy many HD core symptoms including circadian dysfunctions. Because circadian dysfunction manifests early in the disease in both patients and mouse models, we sought to determine if early intervention that improve circadian rhythmicity can benefit HD and delay disease progression. We determined the effects of time-restricted feeding (TRF) on the Q175 mouse model. At six months of age, the animals were divided into two groups: ad libitum (ad lib) and TRF. The TRF-treated Q175 mice were exposed to a 6-h feeding/18-h fasting regimen that was designed to be aligned with the middle of the time when mice are normally active. After three months of treatment (when mice reached the early disease stage), the TRF-treated Q175 mice showed improvements in their locomotor activity rhythm and sleep awakening time. Furthermore, we found improved heart rate variability (HRV), suggesting that their autonomic nervous system dysfunction was improved. Importantly, treated Q175 mice exhibited improved motor performance compared to untreated Q175 controls, and the motor improvements were correlated with improved circadian output. Finally, we found that the expression of several HD-relevant markers was restored to WT levels in the striatum of the treated mice using NanoString gene expression assays.

## Visual Abstract

**Figure:**
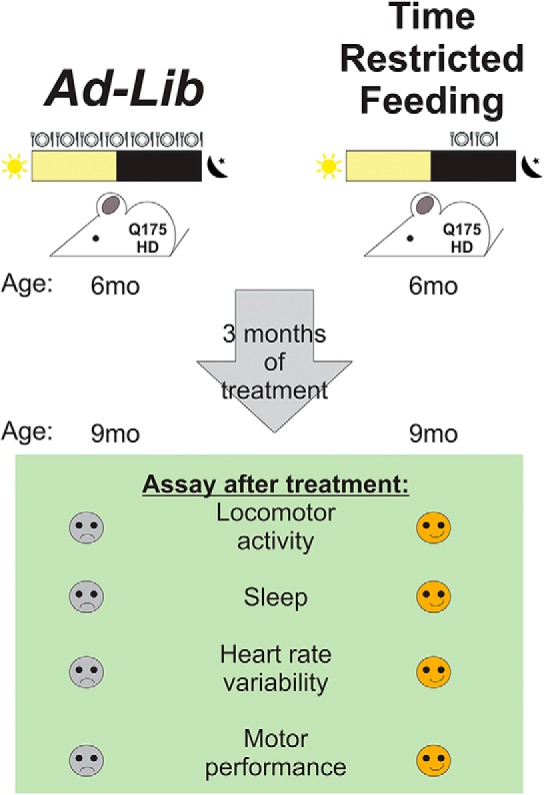

## Significance Statement

Huntington’s disease (HD) is a genetically caused disease with no known cure. Lifestyle changes that not only improve the quality of life but also delay disease progression for HD patients are greatly needed. In this study, we found that time-restricted feeding (TRF) improves activity/rest rhythms in the Q175 mouse model of HD. This treatment also improved motor performance and heart rate variability (HRV) in the HD mice. Finally, TRF altered the expression of HD relevant markers in the striatum. Our study demonstrates the therapeutic potential of circadian-based treatment strategies in a preclinical model of HD.

## Introduction

Huntington’s disease (HD) is caused by an expanded CAG repeat within the first exon of the Huntingtin (*Htt*) gene. The mutated HTT protein leads to dysfunction of a large range of cellular processes, including cytoskeletal organization, metabolism, and transcriptional activities (Bourne et al., 2006; Grimbergen et al., 2008; Fisher et al., 2014). As result, HD patients suffer from progressive neurodegeneration that inflicts cognitive, psychiatric, cardiovascular, and motor dysfunction. The genetic components greatly determine the age of symptom onset and the severity. Generally, the longer the CAG repeat, the earlier the age of onset and the greater the severity of the symptoms (Langbehn et al., 2010). Still, even among patients with the same CAG repeat length, large variabilities in the onset of symptoms (around a decade) and their severity have been reported (Gusella et al., 2014). In addition, studies have shown that environmental factors also affect the disease progression (Wexler et al., 2004). Those reports raise the possibility of environmental modifiers to the disease and suggest that lifestyle changes can increase the health span of the patients. This possibility is important to pursue as there are no known cures for HD.

Disturbances in the timing of sleep, typified by frequent bedtime awakenings, prolonged latency to fall asleep, and more naps during the awake phase, are extremely common in HD and often become apparent years before the onset of classic motor symptoms (Cuturic et al., 2009; Aziz et al., 2010a; Goodman et al., 2011). Similarly, mouse models of HD also exhibit a disrupted circadian rest/activity cycle that mimics the symptoms observed in human patients (Morton et al., 2005; Kudo et al., 2011; Loh et al., 2013). This body of work supports the hypothesis that circadian dysfunctions may interact with HD pathology and exacerbate the symptoms. To test this hypothesis, we have been using the Q175 knock-in model of HD. In previous work (Loh et al., 2013), we have characterized the impact of age (3, 6, 9, and 12 months) and gene dosage (Het and Hom) on the degradation of circadian rhythms in locomotor activity and other HD core symptoms. Recently, a detailed RNA-seq analysis of striatum, cortex, and liver of the Q175 line has been published (Langfelder et al., 2016); therefore, we have a good understanding of the transcriptional changes that occur with age in this model. Finally, recent work has carefully characterized age-related changes in the electroencephalogram (EEG) in both Hom and Het Q175 (Fisher et al., 2016). This wealth of data makes the Het Q175 an ideal preclinical model to examine the impact of circadian interventions on disease trajectory.

The central circadian clock responsible for the generation of daily rhythms is localized in the suprachiasmatic nucleus (SCN) in the hypothalamus. While lighting conditions are a critical environmental input to this timing system, a body of recent work has lead us to appreciate that the feed/fast cycle is also a powerful regulators of the circadian system (Hamaguchi et al., 2015). While progressive, age-related SCN dysfunction has been reported in HD mouse models (Bartlett et al., 2016), a time-restricted feeding (TRF) regimen promises therapeutic potential and can benefit even SCN-lesioned mice (Hara et al., 2001; Mulder et al., 2014). For example, mice under TRF consume equivalent calories from a high-fat diet as those with ad libitum (ad lib) access yet are protected against obesity, hyperinsulinemia, and inflammation and have improved motor coordination (Hatori et al., 2012). In the present study, we examined the impact of imposing a 6-h feeding/18-h fasting regimen that was aligned to the middle [zeitgeber time (ZT) 15-21] of the period when mice normally active (ZT 12-24). The treatment was applied to Q175 Hets starting when the mutants were six months of age and ending when they were nine months. We selected this age range because the Het Q175 start to show disrupted sleep/wake cycles and motor symptoms are just beginning.

## Materials and Methods

The work presented in this study followed all guidelines and regulations of the UCLA Division of Animal Medicine that are consistent with the Animal Welfare Policy Statements and the recommendations of the Panel on Euthanasia of the American Veterinary Medical Association.

### Animals

The Q175 mice used in this study were males on the C57BL6/J background. They arose from a spontaneous expansion of the CAG repeat in the CAG140 transgenic knock-in line (Menalled et al., 2012). The mice were heterozygous (Het) for the Q175 allele with an average of 189 ± 3 CAG repeats. Mutant mice were obtained from The Jackson Laboratory from a colony managed by the CHDI Foundation. The animals were singly housed within light-tight chambers with independently controlled lighting conditions: 12 h of light followed by 12 h of dark (12/12 h LD). The chambers were in the same animal housing facility with controlled temperature and humidity, and each chamber held eight cages of mice, grouped together by feeding treatment. All animals received cotton nestlets, and water was made available at all times. To confirm the effect of timed feeding on daily rhythms and motor performance, we also examined WT mice at nine months of age.

### TRF

Mice were first entrained to a 12/12 h LD cycle for a minimum of two weeks before any treatment. Experimental animals were randomly assigned to one of two feeding conditions: food available ad lib and food available for 6 h during the middle of the active phase during ZT 15-21. By definition, ZT 12 referrers to when the lights go off when the mice are in an LD cycle. Experimental mice were singly housed in cages with a custom made programmable food hopper that could temporally control access to food (Diet Teklad 7013: fat, 18 kcal%; caloric density, 3.13 kcal/g) and prevent food consumption during restricted times. These cages were also equipped with an infrared (IR) motion detector to give us the ability to measure cage activity. The mice were held in these conditions for a total of three months (from six to nine months of age).

### Monitoring of cage locomotor activity

Experimental mice were singly housed in cages with the food hopper as well as IR motion sensors. The locomotor activity recorded as previously described (Wang et al., 2017). Mice were entrained to a 12/12 h LD cycle for a minimum of two weeks before data collection. Locomotor activity data were recorded using Mini Mitter data loggers in 3-min bins, and 10 d of data were averaged for analysis. We used the 10 d of activity data collected just before the motor performance tests during the final two weeks of the TRF schedule. The data were analyzed to determine the period and rhythmic strength as previously described (Loh et al., 2013; Wang et al., 2017). The periodogram analysis uses a χ^2^ test with a threshold of 0.001 significance, from which the amplitude of the periodicities is determined at the circadian harmonic to obtain the rhythm power. The amount of cage activity over a 24-h period was averaged over 10 d and reported here as the arbitrary units (a.u.)/h. The number of activity bouts and the average length of bouts were determined using Clocklab (Actimetrics), where each bout was counted when activity bouts were separated by a gap of 21 min (maximum gap: 21 min; threshold: 3 counts/min). The onset variability was determined using Clocklab by drawing the best-fit line over the 10 d, and averaging the differences between activity onset and best-fit regression of each day.

### Monitoring of immobility-defined sleep behavior

Immobility-defined sleep was determined as described previously (Loh et al., 2013; Wang et al., 2017). Mice were housed in see-through plastic cages containing bedding (without the addition of nesting material) and the food hopper. A side-on view of each cage was obtained, with minimal occlusion by the food bin or water bottle, both of which were top-mounted. Cages were side-lit using IR-LED lights. Video capture was accomplished using surveillance cameras with visible light filters (Gadspot Inc) connected to a video-capture card (Adlink Technology Inc) on a Dell Optiplex computer system. ANY-maze software (Stoelting Co) was used to track the animals.

Immobility was detected when 95% of the area of the animal stayed immobile for >40 s, as was previously determined to have 99% correlation with simultaneous EEG/EMG-defined sleep (Pack et al., 2007; Fisher et al., 2012). Continuous tracking of the mice was performed for a minimum of five sleep-wake cycles, with randomized visits (one to two times per day) by the experimenter to confirm mouse health and video recording. The 3rd and 4th sleep-wake cycles were averaged for further analysis. Immobility-defined sleep data were exported in 1 min bins, and total sleep time was determined by summing the immobility durations in the rest phase (ZT 0-12) or active phase (ZT 12-24). An average wave form of hourly immobile-sleep over the two sleep-wake cycles was produced during the final week of TRF. Variability of awake time was determined using Clocklab to draw the best-fit line over the sleep cycles, and the differences between sleep offset and best-fit regression of each sleep cycle were averaged.

### Rotarod test. Accelerating version

The rotarod apparatus (Ugo Basile) is commonly used to measure motor coordination and balance. This apparatus is, in essence, a small circular treadmill. It consists of an axle or rod thick enough for a mouse to rest over the top of it when it is not in motion and a flat platform a short distance below the rod. The rod is covered with smooth rubber to provide traction while preventing the mice from clinging to the rod. In this study, mice were placed on top of the rubber covered rod. When the mice moved at the pace set by the rotation rate of the rod, they would stay on top of it. When mice no longer move at the selected pace they dropped a short distance to the platform below. The time a mouse remained on the rod, before dropping to the platform was called the latency to fall. Following a 15-min habituation to the testing room, mice were placed on the slowly rotating rod. The rod gradually accelerated from 5 rpm to 38 rpm over the course of the trial. The length of time the mouse stays on the rod was recorded. A two-day protocol for the accelerating rotarod tests was used. On the first day, the mice were trained on the rotarod over five trials. The maximum length of each trial was 600 s, and mice were allowed to rest for a minimum of 60 s between trials. On the second day, mice were tested on the rotarod and the latency to fall from the rotarod was recorded from five trials. Mice were again allowed to rest for a minimum of 60 s between trials. Data from each mouse were analyzed after averaging the times from all five trials. The apparatus was cleaned with 70% alcohol and allowed to dry completely between trials. A dim red-light (2 lux) was used for illumination during active phase testing (night).

### Challenging beam test

The challenging beam test is a modified version of the beam traversal test first described by Goldberg and colleagues (Goldberg et al., 2003), and was used to characterize the motor deficits of Q175 mutant mice in previous studies (Loh et al., 2013, Wang et al., 2017). The beam narrows in four intervals from 33 mm > 24 mm > 18 mm > 6 mm, with each segment spanning 253 mm in length. Apparatus and methods used are similar to those described by Fleming and colleagues (Fleming et al., 2013). The home cage of each mouse is put on the end of the beam as the motivating factor. In this study, animals were trained on the beam for five consecutive trials on two consecutive days. During each trial, each mouse was placed on the widest end of the beam and allowed to cross with minimal handling by the experimenter. On the testing day, a metal grid (10 × 10-mm spacing, formed using 19-gauge wire) was overlaid on the beam. This overlaid grid increased the difficulty of the beam traversal task and provided a visual reference for foot slips made while crossing the grid. Each mouse was subjected to five consecutive trials, which were recorded by a camcorder under dim red-light conditions (2 lux), supplemented with IR lighting for video recording. The videos were scored *post hoc* by two independent observers for the number of missteps (errors) made by each mouse. The observers were masked as to the treatment group of the mice that they were scoring. An error was scored when any foot dipped below the grid. The number of errors was averaged across the five trials per mouse to give the final reported values. The apparatus was cleaned with 70% alcohol and allowed to dry completely between trials. A dim red-light (2 lux) was used for illumination during active phase testing (night).

### Automatic outputs. Core body temperature (CBT), heart rate (HR), and HR variability (HRV)

For the telemetry measurements, methods employed were similar to those previously described (Schroeder et al., 2016; Cutler et al., 2017). Two groups (ad lib and TRF) of Het Q175 mice (*n* = 7/group) were surgically implanted with a wireless radio-frequency transmitter (ETA-F20, Data Sciences International). Mice were singly housed in cages with the food hopper. Cages were placed atop telemetry receivers (Data Sciences International) in a light and temperature-controlled chamber. Standard rodent chow was provided for both groups. Data collection began two weeks after surgery. HR was extrapolated from ECG waveforms using the RR interval.

Data collection and analysis were performed as described previously (Cutler et al., 2017). Data were extracted in 20-s intervals then filtered to remove extreme noise. Remaining valid data segments were averaged into 1-h bins across the 24-h cycle. Mean normal to normal intervals (NN, in ms) and SD of all NN intervals (SDNN, in ms) were calculated for the time domain analysis.

### NanoString analysis of gene expression

Tissue collection and data analysis were performed as described previously (Wang et al., 2017). Four weeks after the final behavioral tests were performed, the Q175 mutants were anesthetized with isoflurane before dissection of the striatum at ZT 15. The brain tissue samples were flash frozen and stored at −80°C before NanoString analysis. The NanoString analysis was performed by LabCorp using a custom CodeSet designed to interrogate 100 transcripts previously implicated in transcriptional changes in the striatum of Q175 mice (Langfelder et al., 2016). The signal intensity of individual genes was normalized by adjusting to internal positive standards within each sample. Eight housekeeping genes were included in the CodeSet: *Gins1, Myh15, Pank2, Poc1b, Pum2, Slc25a15, Ssrp1, and Utp3*. The expression levels for each probe within a sample were scaled using the geometric mean of the eight housekeeping genes for each sample. Each mouse was an individual sample as tissue did not need to be pooled. The fold change of signal intensity was derived by comparing the normalized means between the ad lib group and the TRF group.

### Pathway analysis

To study the HD-changed gene expression data in the context of biological networks, the gene expression data of TRF-treated Q175 and untreated Q175 control samples were analyzed with the Ingenuity Pathway Analysis (IPA) system (Ingenuity Systems). Datasets containing gene identifiers and corresponding expression values were uploaded in the application. Each gene identifier was mapped to its corresponding gene object in Ingenuity Pathways Knowledge. A cutoff of corrected *p* value (i.e., *q* value = 0.005) was set to identify genes whose expression was significantly different as a result of the treatment. These genes were overlaid onto a global molecular network developed from information contained in the Ingenuity Pathways Knowledge Base. Functional analysis using the IPA program identified the biological functions that were most significant to the dataset (uncorrected Fisher’s exact test *p* < 0.05).

### Statistical analysis

We were interested in determining if TRF can delay the progression of symptoms in the Q175 mouse model; Therefore, treated Q175 mice (TRF group) were compared to age-matched untreated Q175 mice (ad lib group) in all experiments. The sample size per group was determined by both our empirical experience with the variability in the prior measures in the Q175 mice (Loh et al., 2013) and a power analysis (SigmaPlot, SYSTAT Software) that assumed a power of 0.8 and an α of 0.05. For the behavioral measures, the analysis was done by two observes masked as to the experimental condition and their values averaged. To assess the impact of TRF after three months, we applied a *t* test for the analysis. To determine the impact of the treatment on temporal activity, sleep, CBT, HR, and HRV waveforms, we used a two-way repeated measures ANOVA (two-way RM ANOVA) with treatment and time as factors. To determine the impact of the treatment on errors made in each beam of the challenging beam test, we used a two-way ANOVA with treatment and beam # as factors. *F* values are reported as *F* (degrees of freedom between groups, degrees of freedom within groups). Pairwise multiple comparison procedures were made using the Holm–Sidak method. Correlations between circadian parameters and motor performance were examined by applying Pearson correlation analysis. Statistical analysis was performed using SigmaPlot. The dataset was examined for normality (Shapiro–Wilk test) and equal variance (Brown–Forsythe test). The power of the statistical tests is reported in Table 1. Between-group differences were determined significant if *p* < 0.05. All values are reported as group mean ± SEM.

**Table 1.** List of distribution, statistical test, and power for each dataset analyzed in this study

Letter	Data structure	Type of test	Power
a food consumption	Normal distribution	*t* test	0.052
b body weight	Normal distribution	*t* test	0.050
c power	Normal distribution	*t* test	0.956
d onset	Normal distribution	*t* test	0.536
e cage activity	Normal distribution	*t* test	0.843
f bout #	Normal distribution	*t* test	0.605
g waveform	Normal distribution	Two-way ANOVA	Time 1.000
			Treatment 0.843
h bout duration	Normal distribution	*t* test	0.729
i bout #	Normal distribution	*t* test	0.413
j sleep waveform	Normal distribution	Two-way ANOVA	Time 1.000
			Treatment 0.179
K sleep duration	Normal distribution	*t* test	0.050
l bout #	Normal distribution	*t* test	0.328
m bout duration	Normal distribution	*t* test	0.895
n wake time onset	Normal distribution	*t* test	0.944
o cycle to cycle	Normal distribution	*t* test	0.440
p daytime activity	Normal distribution	*t* test	0.884
q activity waveform	Normal distribution	Two-way ANOVA	Time 1.000
			Treatment 0.997
r average CBT	Normal distribution	*t* test	0.529
s CBT waveform	Normal distribution	Two-way ANOVA	Time 1.000
			Treatment 0.729
t HR average	Normal distribution	*t* test	0.382
u HR amplitude	Normal distribution	*t* test	0.560
v HR waveform	Normal distribution	Two-way ANOVA	Time 1.000
			Treatment 0.895
w average HVR	Normal distribution	*t* test	0.632
x HRV waveform	Normal distribution	Two-way ANOVA	Time 1.000
			Treatment 1.000
y rotarod	Normal distribution	*t* test	0.911
z beam errors	Normal distribution	*t* test	0.989
aa error by beam	Normal distribution	Two-way ANOVA	beam 1.000
			Treatment 1.000

The first column lists the superscript lowercase letter referring to statistical test in the Results section. The second column is the structure of the data (normal distribution or non-normal). Each of the datasets was examined for normality (Shapiro–Wilk test) and equal variance (Brown–Forsythe test). The third column lists the statistical test. The fourth column gives the observed power value of the statistical test calculated from the actual data.

## Results

By using the programmable food hopper, we could temporally control access to food (ZT 15-21) and prevent food consumption for the rest of the daily cycle. During this 6-h interval, the mice would eat as much as they wanted and the amount of food consumed daily did not vary between the Het Q175 groups (ad lib: 2.8 ± 0.4 g; TRF: 2.8 ± 0.2 g, *t*_(14)_ = −0.13, *p* = 0.900, *t* test^a^). At the time when we performed the recordings and motor assays, the body weights were not different in Q175 mice under TRF compared to age-matched controls (ad lib: 23.9 ± 0.4 g; TRF: 24.5 ± 0.4 g, *t*_(14)_ = −1.03, *p* = 0.320, *t* test^b^).

### TRF increased the amplitude of diurnal rhythms in Het Q175 line

**Figure 1.**
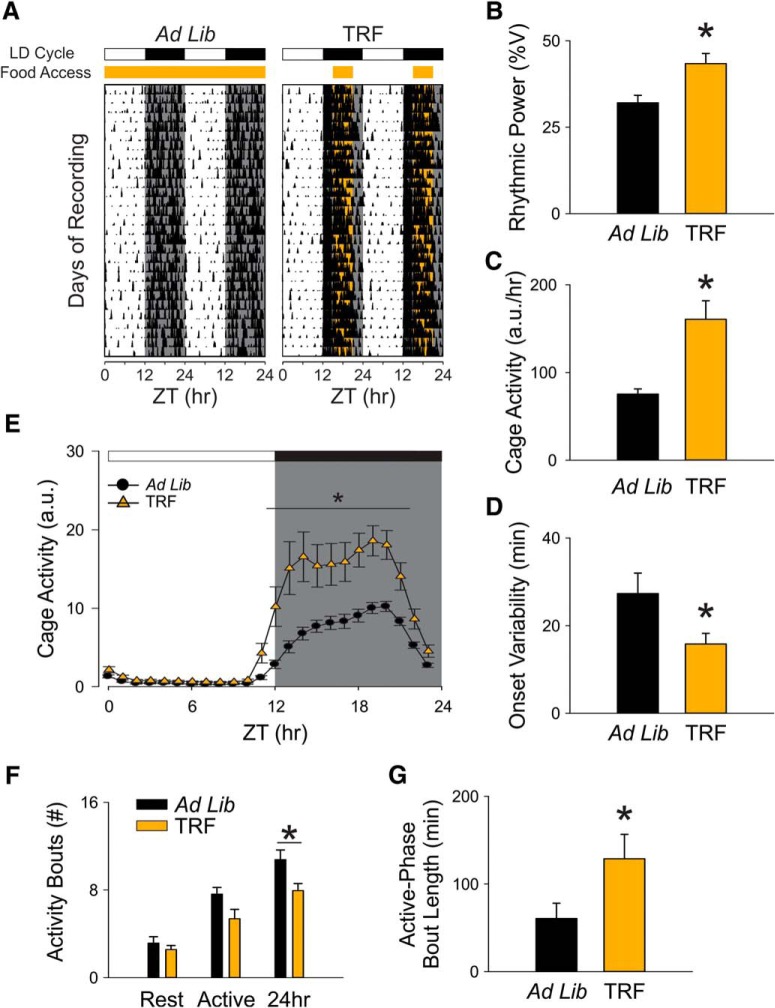
Locomotor activity rhythms were improved by the TRF regimen. ***A***, Examples of cage activity rhythms recorded from Q175 mutants under control (left) and TRF (right) conditions. The activity levels in the actograms were normalized to the same scale (85% of the maximum of the most active individual). Each row represents two consecutive days, and the second day is repeated at the beginning of the next row. The orange bar on the top of actograms indicates the time when food hopper is opened. ***B***, The strength of the activity rhythm is indicated by the power (%V) of the χ^2^ periodogram analysis. ***C***, The averaged level of cage activity. ***D***, The averaged variation in onset from the best-fit regression line. ***E***, Average waveforms from 10 d of cage activity (1-h window) are shown and SEs across animals are indicated. ***F***, The number of activity bouts (separated by a gap of 21 mins or more) during rest phase (ZT 0-12), active phase (ZT 12-24), and 24 h are reported as the level of fragmentation of the circadian activity cycle. Black bars represent Q175 mutants under ad lib condition, and orange bars represent Q175 mutants under timed feeding condition. ***G***, The average length of activity bouts during their active phase. The white/black bar on the top of actograms (***A***) and waveforms (***E***) indicates the 12/12 h LD cycle. The temporal activity wave form was analyzed using a two-way RM ANOVA with time and treatment as factors. Other comparisons between Q175 cohorts were made using a *t* test. Asterisks represent significant differences due to TRF regimen compared to ad lib controls (*p* < 0.05); *n* = 8/group.

At early disease stage (nine months of age), the TRF-treated group showed greatly improved circadian locomotor activity rhythms (Fig. 1*A–D*), evidenced by the stronger rhythmic power (ad lib: 32.1 ± 2.2; TRF: 43.4 ± 2.9, *t*_(14)_ = −3.12, *p* = 0.008, *t* test^c^) and lower activity onset variability (ad lib: 27.3 ± 4.6 min; TRF: 15.8 ± 2.4 min, *t*_(14)_ = 2.2, *p* = 0.045, *t* test^d^) than the control group. The amount of cage activity was also increased under the TRF regimen (ad lib: 75.3 ± 5.9 a.u./h; TRF: 160.7 ± 21.1 a.u./h, *t*_(14)_ = 42, *p* = 0.005, *t* test^e^). These increases in rhythm power and activity amount coincided with a decreased total number of activity bouts (ad lib: 10. 8 ± 0.9; TRF: 7.9 ± 0.6, *t*_(14)_ = 2.6, *p* = 0.021, *t* test^f^). A temporal activity wave form indicated more robust activity levels in the TRF-treated group at night when the mice should be active (Fig. 1*E*). A two-way RM ANOVA^g^ revealed a significant effect of time (*F*_(23,382)_ = 70.07, *p* < 0.001), treatment (*F*_(1,14)_ = 10.82, *p* = 0.005), and a significant interaction between the two factors (*F* = 8.24, *P* < 0.001). A further examination of activity bouts at night (ZT 12-24) revealed that the TRF group had longer bout lengths (ad lib: 60.6 ± 17.5 min; TRF: 128.8 ± 27.8 min, *t*_(14)_ = 48, *p* = 0.038, *t* test^h^) without a significant increase in the number (ad lib: 7.6 ± 0.6; TRF: 5.4 ± 0.9, *t*_(14)_ = 2.15, *p* = 0.05, *t* test^i^), suggesting that the robust amplitude of diurnal rhythms in the TRF group was due to the consolidated and high amount of locomotor activity during the active phase (Fig. 1*F*,*G*). Under TRF, the activity parameters in the Q175 mice were no longer significantly different from WT (Tables 1, 2). These findings demonstrate that TRF treatment significantly improved the activity rhythms of the HD mutant mice.

**Table 2.** Comparisons of age-matched WT under ad lib conditions to Q175 mice under ad lib or TRF regimen (*n* = 8/group)

	WT ad lib	WT ad lib vs Q175 ad lib		WT ad lib vs Q175 TRF	
Locomotor activity rhythm	AVG ± SEM	Difference	*p* value	Difference	*p* value
Rhythmic power (V%)	>32.59 ± 2.12	3.93	0.234	−10.82	**0.009**
Cage activity (a.u/h)	152.47 ± 19.08	75.67	**0.002**^U^	−8.23	0.7
Onset variability (min)	23.20 ± 2.84	−4.13	0.461	7.41	0.068
Bouts/d	8.44 ± 0.39	−2.34	**0.007**	0.50	0.517
Average bout length (rest-phase)	166.82 ± 22.33	106.20	**0.002**	38.01	0.305
Sleep behavior rhythm					
Daily sleep	665.42 ± 16.28	−57.12	0.081	−20.89	0.534
Bouts/d	8.44 ± 0.79	0.25	0.779	−0.88	0.443
Average bout length (night)	85.54 ± 21.52	−74.83	0.075	20.03	0.721
Awake time (ZT)	12.03 ± 0.1	−0.60	**0.002**^U^	0.10	0.329
Awake deviation time I (min)	13.62 ± 3.26	−24.07	**0.004**	−5.70	0.382
Motor performance					
Latency to fall (s)	320.65 ± 24.37	64.65	0.119	−99.4	**0.028**
Crossing errors (#)	3.09 ± 0.21	−4.35	**<0.001**	−1.88	**0.002**^U^

The results of *t* tests are reported if data passed normality tests. DF = 14. For parameters that did not pass normality tests, the Mann–Whitney rank-sum test was run and the *U* statistic reported; *p* < 0.05 was considered significant. In this and subsequent tables significant differences are shown in bold.

### TRF shifted the timing but not the total amount of sleep behavior in the Het Q175 mice

**Figure 2.**
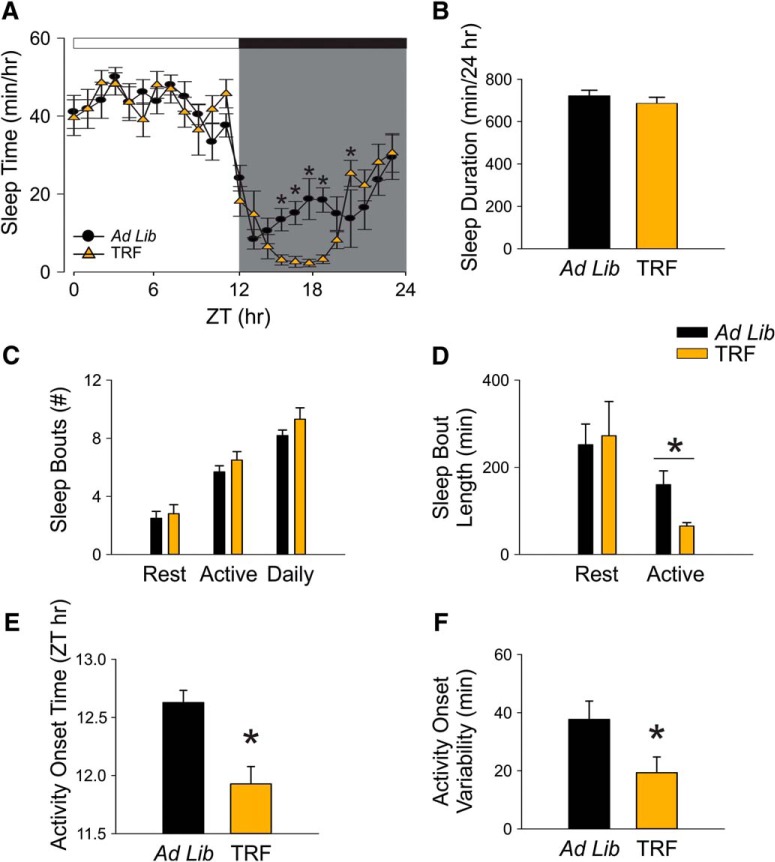
TRF prevented disease-caused awakening time without altering the amount of sleep behavior. Video recording in combination with automated mouse tracking analysis software was used to measure immobility-defined sleep. ***A***, Running averages (1-h window) of immobility-defined sleep in Q175 mutants with ad lib (black) and timed feeding (orange) are plotted. The white/black bar on the top of wave form indicates the 12/12 h LD cycle. ***B–F***, Quantification of the immobility-defined sleep rhythms. The temporal sleep wave form was analyzed using a two-way RM ANOVA with time and treatment as factors. Other comparisons between Q175 cohorts were made using a *t* test. Asterisks represent significant differences due to TRF regimen compared to ad lib controls (*p* < 0.05); *n* = 8/group.

The immobility-defined sleep behavior was measured using video recording in combination with automated mouse tracking analysis software. During the 6 h when food was available at night, the TRF-treated Q175 mice slept less than untreated Q175 controls (Fig. 2*A*). A two-way RM ANOVA^j^ was used to analyze the temporal pattern of sleep (1-h bins) of each group. The analysis revealed significant effect of time (*F*_(23,382)_ = 36.575, *p* < 0.001) and significant interaction between time and treatment (*F*_(23)_ = 2.23, *p* = 0.002), but the effect of treatment did not reach a significant level (*F*_(1,14)_ = 2.033, *p* = 0.155). No significant changes were detected in the total amount of sleep time over a 24-h cycle (ad lib: 722.5 ± 25.6 min; TRF: 686.3 ± 28.4 min, *t* = 0.95, *p* = 0.36, *t* test^k^; Fig. 2*B*). No significant difference was found in the total number of sleep bouts over a 24-h cycle (ad lib: 8.2 ± 0.4; TRF: 9.3 ± 0.8, *t*_(14)_ = 58, *p* = 0.33, *t* test^l^). The sleep bouts at night were significantly shorter in the TRF group than the control group (ad lib: 160.4 ± 31.6; TRF: 65.5 ± 7.9, *t*_(14)_ = 93, *p* = 0.007, *t* test^m^), suggesting that TRF group had shorter naps than the control group in their active phase (Fig. 2*C*,*D*).

The TRF treatment advanced the phase when the Q175 mice transitioned from sleep to awake states (ad lib: ZT 12.6 ± 0.2 h; TRF: ZT 11.9 ± 0.1 h, *t*_(14)_ = 3.84, *p* = 0.002, *t* test^n^; Fig. 2*E*). The TRF group also exhibited a more precise awakening time than the Q175 control mutants (ad lib: 37.7 ± 6.3 min; TRF: 19.3 ± 5.4 min, *t*_(14)_ = 2.21, *p* = 0.044, *t* test^o^; Fig. 2*F*). Under TRF, the beginning of activity and the cycle-to-cycle variability in sleep behavior in the Q175 mice were no longer significantly different from WT (Tables 2, 3). Overall, these findings demonstrate that the TRF regimen improved sleep behavior in Q175 mice.

**Table 3.** Comparisons of age-matched WT under ad lib to regimen (*n* = 8/group)

	WT TRF	WT TRF vs WT ad lib	
Locomotor activity rhythm	AVG ± SEM	Difference	*p* value
Rhythmic power (V%)	57.03 ± 3.15	24.44	**<0.001**
Cage activity (a.u/h)	269.96 ± 20.24	117.49	**<0.001**
Onset variability (min)	31.54 ± 2.49	8.34	**0.028^U^**
Bouts/d	6.8 ± 0.38	−1.64	**0.009**
Average bout length (rest-phase)	202.55 ± 25.87	35.74	0.313
Sleep behavior rhythm			
Daily sleep	646.25 ± 31.61	−19.17	0.598
Bouts/d	9.5 ± 0.61	1.06	0.279
Average bout length (night)	60.06 ± 12.8	−25.47	0.326
Awake time (ZT)	11.90 ± 0.16	−0.12	0.095
Awake deviation time I (min)	19.57 ± 6.04	5.94	0.42
Motor performance			
Latency to fall (sec)	457.08 ± 22.12	136.43	**<0.001**
Crossing errors (#)	3.28 ± 0.31	0.19	0.6
Body weight (g)	29.02 ± 0.87	−0.76	0.343

Find the values of ad lib in Table 2. The results of *t* tests are reported if data passed normality tests. DF = 14. For parameters that did not pass normality tests, the Mann–Whitney rank-sum test was run and the *U* statistic reported; *p* < 0.05 was considered significant.

### TRF improved autonomic outputs in the Het Q175 mice

**Figure 3.**
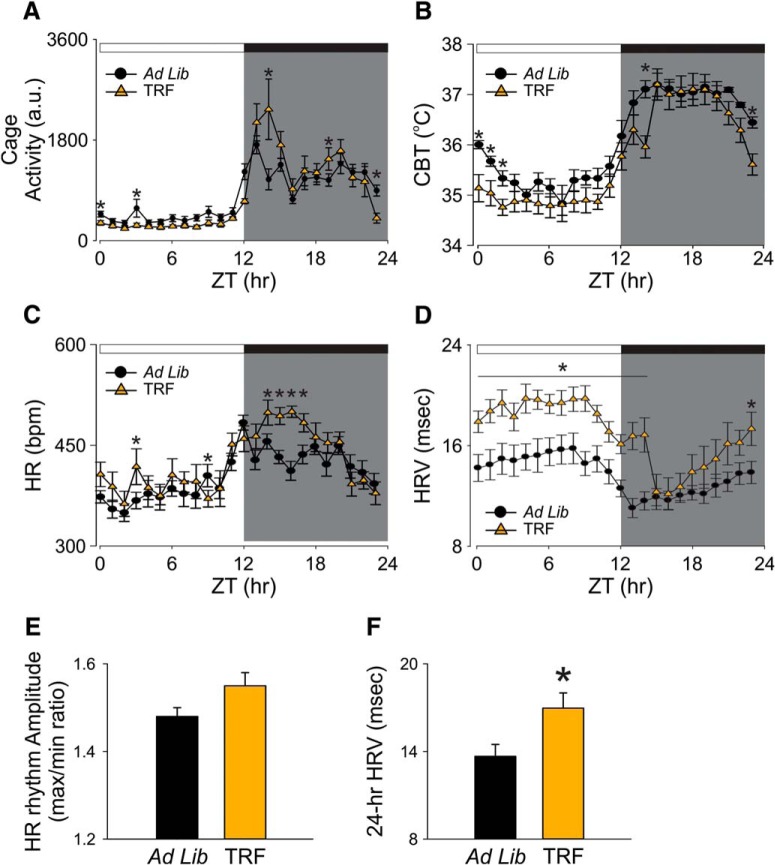
Autonomic output rhythms were improved by the TRF regimen. The autonomic outputs from ad lib (black circles) and TRF (orange triangles) Q175 mice were recorded simultaneously using telemetry device. ***A–D***, Hourly running averages of activity (***A***), CBT (***B***), HR (***C***), and HRV from both groups are plotted (***D***). ***E***, The HR rhythm amplitude, determined by the ratio of max and min of the day, in control and TRF-treated Q175 mice. ***F***, The 24-h averaged HRV in control and TRF-treated Q175 mice. The temporal waveforms of autonomic outputs were analyzed using a two-way RM ANOVA with time and treatment as factors. Other comparisons between Q175 cohorts were made using a *t* test. Asterisks represent significant differences due to TRF regimen compared to ad lib controls (*p* < 0.05); *n* = 7/group.

It has been shown that dysfunction in the circadian regulation of autonomic outputs can be detected early in disease progression in the Q175 mice (Cutler et al., 2017). In the present study, we measured the impact of TRF on activity, CBT, HR, and HRV measured simultaneously in freely moving Q175 mice (Fig. 3). The TRF Q175 mice exhibited higher levels in activity, CBT, and HR at some phases of the daily cycle (Fig. 3*A–C*). TRF also reduced the inappropriate activity during the daytime (ZT 0-12) when mice are normally less active (ad lib: 618.6 ± 96.6 a.u.; TRF: 308.4 ± 33.9 a.u., *t*_(12)_ = 3.03, *p* = 0.010, *t* test^p^). A two-way RM ANOVA^q^ was applied on the activity wave form and significant effects of time (*F*_(23,334)_ = 21.86, *p* < 0.001), treatment (*F*_(1,12)_ = 23.81, *p* < 0.001) and interaction (*F*_(23)_ = 3.68, *p* < 0.001) were detected. In addition, the daily 24-h averaged CBT was not significantly different between the two groups (ad lib: 37.1 ± 0.1°C.; TRF: 36.7 ± 0.3°C, *t*_(12)_ = 3.03, *p* = 0.17, *t* test^r^). The TRF-treated group showed a lower CBT at the dark/light transition (ZT 23-2; Fig. 3*B*). A two-way RM ANOVA^s^ confirmed significant effects of time (*F*_(23,334)_ = 28.64, *p* < 0.001) and treatment (*F*_(1,12)_ = 7.65, *p* = 0.006) without an interaction between the two factors (*F*_(23)_ = 1.05, *p* = 0.398). Despite no difference in the daily 24-h averaged HR (ad lib: 405. 9 ± 8.0 bpm; TRF: 424.1 ± 10.2, *t* = −1.4, *p* = 0.190, *t* test^t^), the amplitude of the rhythm (max/min ratio) was improved by the TRF regimen (ad lib: 1.5 ± 0.02 bpm; TRF: 1.6 ± 0.03 bpm, *t* = −2.18, *p* = 0.049, *t* test^u^; Fig. 3*E*). The TRF group exhibited higher HR (ZT 13-17) when the food was available. As measured by two-way ANOVA^v^, significant effects of time (*F*_(23,334)_ = 10.21, *p* < 0.001) and treatment (*F*_(1,12)_ = 11.39, *p* < 0.001) were detected. But no interaction between the two factors (*F*_(23)_ = 1.52, *p* = 0.06) was detected. Finally, the TRF-treated group exhibited higher levels in HRV in the rest phase as well as the beginning of active phase than the Q175 control group (Fig. 3*D*). The TRF-treated Q175 mice had significantly higher 24-h averaged HRV than the control Q175 mice (ad lib: 13.7 ± 0.8 msec.; TRF: 17.0 ± 1.0 msec, *t*_(12)_ = −2.5, *p* = 0.028, *t* test^w^). A two-way RM ANOVA^x^ confirmed significant effect of time (*F*_(23,334)_ = 8.23, *p* < 0.001) and treatment (*F*_(1,12)_ = 39.6, *p* < 0.001) without a significant interaction (*F*_(23)_ = 1.33, *p* = 0.140). Overall, the TRF regimen improved the daily rhythms in physiologic, autonomically-driven outputs.

### TRF improved motor performance in the Het Q175 mice

**Figure 4.**
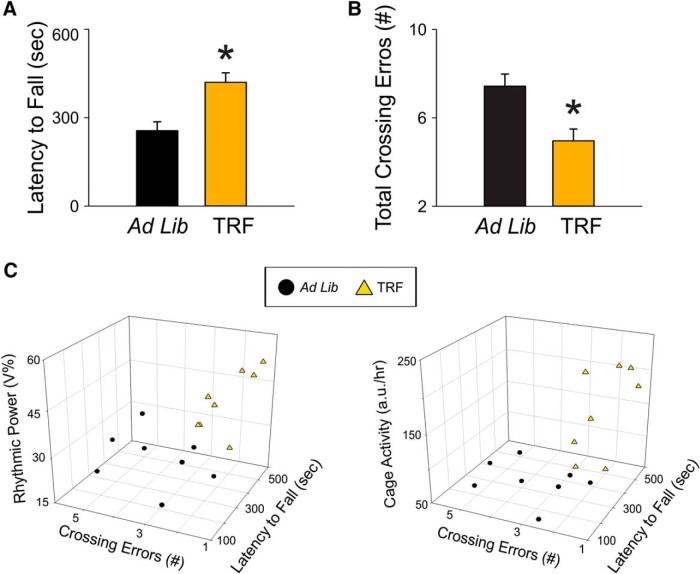
TRF improved motor performance in the Q175 HD model. ***A***, The accelerating rotarod test revealed that the TRF treatment improved motor performance by showing longer latency to fall. ***B***, The challenging beam motor test indicated that the TRF treatment improved performance (fewer errors) by making fewer errors when the mice crossed the balanced beam. ***C***, The circadian parameters and the performance in the two motor tests of individual mouse in ad lib group (black circles) and TRF group (orange triangles) are plotted in a 3D-XYZ grid. In this XYZ grid, there are two distinctive clusters, suggesting that the mouse with stronger circadian rhythms performed better in both motor tests. Comparisons between Q175 cohorts were made using a *t* test. Asterisks represent significant differences due to TRF regimen compared to ad lib controls (*p* < 0.05). The correlations between circadian parameters and motor performance are described in the text; *n* = 8/group.

One of the defining symptoms of HD is the incidence of movement disorders in early-stage patients and we hypothesized that TRF may improve the motor symptoms. To test this hypothesis, we assessed motor performance using two tests that have been shown to detect motor coordination deficits in Q175 mice: the accelerating rotarod (Fig. 4*A*) and challenging beam tests (Fig. 4*B*). The Q175 mice on TRF had a longer latency to fall compared to age-matched Q175 ad lib-fed mutants (ad lib: 256 ± 30.4 min; TRF: 420.1 ± 32.2 min, *t*_(14)_ = −3.7, *p* = 0.002, *t* test^y^). In addition, the treated Q175 mice made significantly fewer errors compared to control Q175 mice (ad lib: 7.4 ± 0.5; TRF: 4.9 ± 0.5, *t*_(14)_ = 3.23, *p* = 0.006, *t* test^z^). Breaking down the errors made by beam width, the two-way ANOVA^aa^ revealed a significant effect of treatment (*F*_(1,14)_ = 15.22, *p* < 0.001), effect of beam width (*F*_(3,62)_ = 26.17, *p* < 0.001), and interaction between the two factors (*F*_(3)_ = 3.924, *p* = 0.013). *Post hoc* analysis indicates that the main difference between treated and control Q175 mice were the errors in the narrowest beam (ad lib: 3.4 ± 0.5; TRF: 1.8 ± 0.2, t = 4.84, *p* < 0.001, *t* test).

The TRF-treated Q175 mice which showed the most improved circadian output also had better performance in the two motor tests (Fig. 4*C*). In a XYZ grid composed of key activity rhythms parameters and performance of motor tests, there were two distinctive clusters which indicated that the mice with improved locomotor activity rhythm performed better in both motor tests. The correlation analysis indicated that the rhythmic power tended to be positively correlated with the amount of time staying on the accelerating rotarod (coefficient = 0.54, *p* = 0.17) and was negatively correlated with numbers of errors made crossing the narrowest beam (coefficient = −0.52, *p* = 0.04) in the TRF group. This correlation was not detected in the Q175 control group (coefficient = 0.16 and 0.13, respectively). Similarly, the TRF-treated group showed a negative correlation between their cage activity level and beam crossing errors (coefficient = −0.51, *p* = 0.01). This correlation was, again, not detected in the Q175 control group (coefficient = −0.06). These data indicate that the TRF-driven improvement in activity rhythms is correlated with the reduction in beam crossing errors.

### Expression of multiple HD markers in striatum were altered by TRF

**Figure 5.**
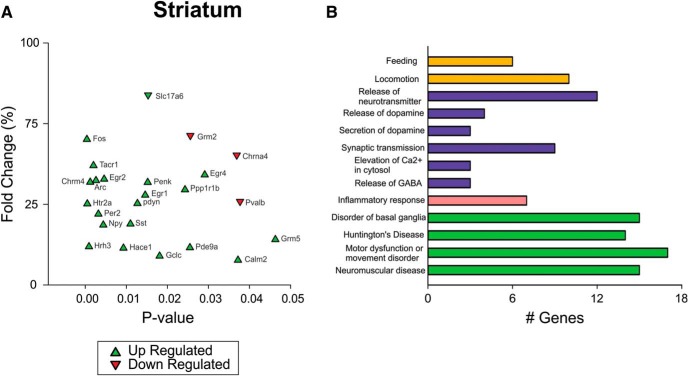
Altered expression level of multiple HD markers in the striatum of the Q175 HD model. ***A***, Differentially expressed genes in the striatum observed between TRF group and ad lib group using NanoString (find all gene expression data in Table 6). The same Q175 mice that underwent activity/sleep monitoring and behavioral tests were allowed to recover for four weeks from manipulations before tissue collection. The signal intensity of individual genes was normalized by adjusting to internal positive standards within each sample (see Materials and Methods). ***B***, Enriched functional clustering in the striatum using the IPA analysis tool (based on data in Table 6; uncorrected Fisher’s exact test *p* value < 0.05). The clusters of interest with statistical significance are picked and enriched biofunctions in those picked clusters are shown (in descending order of significance). The picked clusters include Behavior (*p* = 2.72E-17, color orange), Cell-to-cell signaling and interaction (*p* = 1.02E-17, color blue), inflammatory response (*p* = 2.87E-04, color pink), and neurologic disease (*p* = 8.74E-14, color green).

Striatum is one of the key brain structures of the cortical-basal ganglia circuit controlling motor function, and it has been shown to be particularly vulnerable in HD. Previous work has identified HD-driven changes in transcription in the striatum of the Q175 mouse (Langfelder et al., 2016). Using NanoString technology, we examined the impact of TRF on changes in gene expression of HD markers in the striatum of the Q175 mice as previously described (Wang et al., 2017). The expression patterns were compared to Q175 ad lib controls (Table 4). The TRF regimen altered expression of immediate early genes such as *Arc*, *Erg1,2,4*, and *Fos*, as well as receptors for neurotransmitters such as acetylcholine, histamine, 5HT, tachykinin, and dynorphin (Fig. 5*A*). The IPA analysis tool was applied to the total dataset (Table 5) to identify corresponding enriched pathways and biofunctions (Table 6). The top canonical pathways identified included (in descending order of significance): G protein-coupled receptor (GPCR) signaling, cAMP-mediated signaling, and glutamate receptor signaling. The top upstream regulators included BDNF, CREB1, and HTT. Hence, the TRF treatment significantly altered the patterns of expression of genes linked to HD and modulated multiple transcriptional pathways.

**Table 4.** Top 5 HD markers in the striatum of Q175 altered by the TRF treatment

Comparison		Q175 vs WT			Ad lib vs TRF	
Age		2 months	6 months o	10 months	9 months	
Gene Symbol	Full name	Log2 fold change			Log2 fold change	*p* value
Striatum						
Fos	FBJ osteosarcoma oncogene	ns	↓	ns	↑	0.0004
Htr2a*	5-Hydroxytryptamine (serotonin) receptor 2A	ns	ns	ns	↑	0.0005
Hrh3	Histamine receptor H3	ns	↓	↓	↑	0.0009
Chrm4	Cholinergic receptor, muscarinic 4	↓	↓	↓	↑	0.0012
Tacr1	Tachykinin receptor 1	ns	↓	↓	↑	0.0020

*P* value of the *t* test comparison with Q175 housed under ad lib is shown. Asterisk indicates HD markers changed in both the striatum and cortex. Transcripts increased by the treatment (Log2 fold change) are shown in green (↑) and those decreased by the treatment in red (↓). Transcripts without significant change (*p* > 0.05) are shown in gray (ns); 24% gene expressions in the striatum and 7% gene expressions in the cortex are altered by the TRF treatment. Among altered genes in striatum, >50% genes (13/24) that are shown downregulated in Q175 controls (comparison with age-matched WT controls (Lengfelder et al., 2016) are upregulated by TRF.

**Table 5.** Top 10 canonical pathways and upregulators identified using IPA analysis in striatum of Q175 under TRF regimen

Ingenuity canonical pathways	−log (*p* value)
G protein-coupled receptor signaling	7.65
cAMP-mediated signaling	6.73
Glutamate receptor signaling	6.08
Neuropathic pain signaling in dorsal horn neurons	5.02
Gαi signaling	4.94
Synaptic long-term potentiation	3.38
Gαq signaling	3.03
iNOS signaling	2.88
CREB signaling in neurons	2.87
Serotonin receptor signaling	2.77
Upstream regulator	−log (*p* value)
BDNF	13.41
CREB1	12.27
Cocaine	11.87
CNTF	11.14
HTT	10.82
TET1	10.40
GDNF	9.74
ADCYAP1R1	9.72
Dalfampridine	8.95
Haloperidol	8.90

**Table 6.** Full dataset of expression of HD markers in the striatum of Q175 that are tested by using NanoString technology. Bold text indicates significant difference between ad lib and TRF feeding protocols

Gene symbol	−Log (*p* value)	Log 2 Fold Change
Aco2	0.51	−0.09
Aif1	0.58	0.13
Apba2bp	0.60	−0.37
Arc	**2.58**	0.11
Bdnf	0.54	−0.56
Bhlhb2	0.16	−0.12
C1qc	0.17	0.14
C3	0.49	0.05
C4a	0.60	0.08
calb1	0.39	0.11
Calm1	0.74	0.03
Calm2	**1.43**	−0.16
Calm3	0.06	−0.09
Cdkn1c	0.04	−0.21
Chat	0.55	0.07
Chga	0.54	−0.01
Chrm1	0.33	−0.13
Chrm4	**2.92**	0.17
Chrna4	**1.43**	−0.15
Chrnb2	0.62	0.07
Cnr1	1.02	0.04
Cth	0.28	0.09
Dnajb5	0.13	−0.04
Drd1a	1.06	0.21
Drd2	1.00	0.25
Egr1	**1.84**	0.13
Egr2	**2.34**	0.24
Egr3	0.86	0.08
Egr4	**1.54**	0.21
F8a	1.24	−0.01
Fos	**3.39**	0.23
Fth1	0.34	0.03
Gabra1	0.43	−0.02
Gabrd	0.05	0.11
Gclc	**1.74**	0.15
Gclm	0.07	−0.08
Gfap	1.03	0.03
Grm2	**1.59**	−0.47
Grm5	**1.33**	0.01
Hace1	**2.03**	−0.03
Hmox1	0.86	0.20
Hrh3	**3.04**	0.31
Htr1a	0.03	−0.36
Htr1b	1.22	0.27
Htr2a	**3.32**	0.15
Htt	0.50	−0.10
Il12b	0.73	0.04
Il6	0.41	−0.16
Kcnip2	1.05	0.10
Lonp1	0.67	0.05
Nfe2l2	0.01	−0.05
Ngf	0.75	−0.26
Nos1	0.96	0.02
Nos3	0.09	0.10
Npy	**2.35**	−0.02
Nqo1	0.90	0.03
Ntrk1	1.18	0.13
Ntrk2	1.12	−0.09
Pde10a	0.97	0.20
Pde9a	**1.59**	0.02
(Continued)		
pdyn	**1.89**	0.22
Penk	**1.82**	0.26
Penk1	**1.80**	0.23
Per2	**2.50**	−0.01
Ppargc1a	0.08	0.05
Ppp1r1b	**1.61**	0.19
Ptpn5	0.76	0.09
Pvalb	**1.42**	0.02
Rgs4	0.09	0.00
Rrs1	0.88	0.16
Ryr1	0.15	−0.14
Sap25	0.72	0.03
Slc17a6	**1.81**	−0.15
Slc17a7	0.10	−0.70
Slc1a2	0.12	−0.09
Slc6a3	0.78	0.16
Slco6b1	0.61	0.41
Snap25	0.12	−0.08
Sod1	1.01	0.01
Sod2	0.00	0.05
Sst	**1.96**	0.17
Tac1	1.09	0.15
Tacr1	**2.71**	0.33
Tfeb	0.98	0.03
Tmsb10	0.05	0.24
Vgf	0.69	0.08
hHTT polypro	0.01	−0.12
mHTT polypro	0.15	−0.01

## Discussion

A range of circadian deficits in the mouse models of HD have been reported, detailing the impact on rhythms in behavior and physiology (Bourne et al., 2006; Ciammola et al., 2006; Grimbergen et al., 2008; Cuturic et al., 2009; Kuljis et al., 2012; Fisher et al., 2016). The findings suggest that the most common sleep-related clinical complaints of HD patients (i.e., difficulty falling asleep, frequent awakenings during sleep, and difficulty staying awake during the active cycle) are due, at least in part, to the disease-induced dysfunction in the circadian system. These findings raise the possibility of treating HD symptoms by improving the regularity/robustness of circadian rhythms in activity and rest (Wang et al., 2017; Whittaker et al., 2017).

In the present study, the Het Q175 mice were allowed access to their food (standard chow, 6 h) nightly for three months starting at an age before the onset of motor symptoms. We confirmed that the animals consumed similar amounts of food and the body weights were not significantly decreased by this feeding regimen. We demonstrate that the nightly TRF regimen improved the daily activity rhythm with increases in the rhythmic strength as measured by power of the periodogram and decreases in cycle-to-cycle variability in activity onset. Prior work in WT mice did not find an impact of TRF on locomotor activity patterns (Hatori et al., 2012). While we are not sure of the difference, we did evaluate older mice (six months) who may be already exhibiting some age-related decline in locomotor activity rhythms. The TRF treatment also advanced the time that the mice ended their sleep phase without changes in total amount of sleep per cycle. Critically, the TRF regimen also improved performance of the HD mutant mice on two different motor tests.

The beneficial impact of TRF on motor performance could be dependent on or independent from the improvements in circadian output. We examined this issue by taking advantage of the animal-to-animal variation in the impact of the treatment on circadian and motor function. Using our most sensitive motor assay (i.e., challenge beam test), we found that the improved circadian behavior was correlated with improved motor function in the TRF group (coefficient = −0.52, *P* = 0.04). This finding leads us to conclude that improved circadian timing underlies the improved motor function in the treated mice. Furthermore, a variety of different approaches aiming to boost circadian output have now been found to improve motor functions in different HD mouse models. There is evidence that improving the sleep/wake cycle with sleep-inducing drugs (Pallier et al., 2007; Kantor et al., 2016), stimulants (Cuesta et al., 2012; Whittaker et al., 2017), bright light and restricted wheel access (Cuesta et al., 2014), and blue light (Wang et al., 2017) can treat HD symptoms. This body of work supports our general hypothesis that TRF improves circadian robustness and acts via this mechanism to delay disease symptoms in HD.

Our data clearly demonstrate that the benefits of TRF extend to physiologic measures such as HRV. Cardiovascular events are a major cause of early death in the HD population (Lanska et al., 1988; Sørensen and Fenger 1992) and the dysfunctional autonomic nervous system may be linked to the increased cardiovascular susceptibility. HRV measures the variation in the beat-to-beat (R-R) interval. It reflects the dynamic balance of sympathetic and parasympathetic control of heart function, and displays a robust circadian rhythm. A prior study demonstrated that the Q175 mice exhibit a loss of circadian control in HRV day/night differences, as well as an overall decrease in HRV over a 24-h period when compared to WT controls (Cutler et al., 2017). It is worthwhile to note that a similar decrease in HRV has also been reported in HD patients beginning during the presymptomatic stage of disease progression (Andrich et al., 2002; Aziz et al., 2010b). Reduced HRV is generally considered an indication of poor cardiovascular health and a predictor for cardiovascular disease and mortality (Thayer et al., 2010). To our knowledge, this is the first study showing that a TRF regimen can improve HRV in a disease model.

Prior work in *Drosophila* has also demonstrated the benefits of TRF in ameliorating age-related cardiovascular decline (Melkani and Panda, 2017). In this model, TRF downregulates expression of gene involved in mitochondrial electron transport while increasing expression of a cytoplasmic chaperonin (Gill et al., 2015). This study also found that mutations in circadian clock genes prevented the benefits of TRF. TRF improved the amplitude of the day/night rhythms in many circadian-regulated transcripts. In mice, genetic disruption of the circadian clock results in a variety of cardiovascular deficits (Paschos and FitzGerald, 2010; Young, 2016). Together, this work suggests that TRF can work in concert with the photic regulation of the circadian system to boast the amplitude and perhaps the phasing of the molecular clock-work.

Lifestyle interventions have been suggested to be preventative and therapeutic for diseases associated with aging, such as type-2 diabetes, cardiovascular disease and increasingly neurodegenerative disorders. For example, caloric restriction (CR) has consistently been found to prolong life span and protect against a variety of pathologic conditions (Heilbronn and Ravussin 2003; Fontana et al., 2004). Conceptually, the TRF regimen used in the present study is quite distinct from CR. While CR focuses on overall, dramatic reduction in energy intake, TRF emphasizes the temporal pattern of fasting without a reduction in overall energy intake. Mechanistically, TRF may activate the same beneficial biochemical pathways as CR (Mattson et al., 2014; Longo and Panda 2016) but would likely be easier to implement in a patient population (Scheen, 2008; Marder et al., 2009). In humans, the time of food availability would be during the day when food is normally consumed while the fast would be extended past the normal night. Prior studies have demonstrated the benefits of an 8:16 feed/fast cycle in improving the metabolic state and motor coordination of mice without altering caloric intake or nutrient composition (Hatori et al., 2012; Chaix et al., 2014). In the HD-N171-82Q mouse model, CR improves motor performance and survival while reducing cell death (Duan et al., 2003). Prior work in the R6/2 HD model has shown that TRF can restore HD-driven disruption in circadian gene expression in the liver (Maywood et al., 2010) and improve locomotor activity as well as exploratory behavior in the open field without increasing life span (Skillings et al., 2014). Together, these data suggest that feeding schedules could play a role in the treatment of HD and could lead to the development of new treatment options for neurodegenerative disorders.

The mechanisms underlying the beneficial effects of the TRF regimen on Q175 mouse model are uncertain and likely mediated by multiple pathways. Our data indicate that the TRF treatment changes the transcriptional environment in a brain region intimately involved in HD, i.e., the striatum. We used the NanoString technology with the IPA platform to analyze the transcriptional changes evoked by TRF. We found that >50% of genes (13/24) that had been shown downregulated in Q175 controls in a prior study (comparison with age-matched WT controls (Langfelder et al., 2016) were upregulated by this treatment (Table 4), suggesting our circadian manipulation may exert beneficial effects through these pathways (Table 5). For example, striatal histamine receptor H3 (*Hrh3*) may connect improved circadian rhythms to improved motor functions. *Hrh3*, a GPCR, is strongly expressed in the cortico-striatal circuits controlling motor behavior (Pollard et al., 1993). Prior work found a signiﬁcant reduction in Hrh3 radioligand binding in tissue of HD patients (Goodchild et al., 1999) suggesting a central role of the histaminergic system in this basal ganglia disorder. Histamine is a well-known regulator of the sleep-wake cycle (Lin et al., 2011; Gondard et al., 2013) and specifically, H3R modulates striatal neurons through its regulation of glutamate (Ellender et al., 2011), GABA (Garcia et al., 1997; Ellender et al., 2011), and dopamine (Schlicker et al., 1993; González-Sepúlveda et al., 2013) release. In a recent study, we found that daily treatment with an H3R inverse agonist improved several behavioral measures in the Q175 mice including activity and sleep rhythms, exploratory behavior, mood (Whittaker et al., 2017). GPCR signaling and glutamate receptor signaling are the top three pathways identified in the IPA analysis as being regulated by TRF. Unfortunately, the feeding schedule did not reduce the levels of mutant *Htt* (Table 6). Nevertheless, identifying treatments that improve the standard of living for HD patients remains an important goal. Future work will need to specifically evaluate the role of the histaminergic system in mediating the benefits of TRF for the sleep-wake cycles as well as motor performance.

## Conclusion

Imposed feeding cycles have the capacity to synchronize or increase the amplitude of circadian oscillations throughout the body. Disturbances in the sleep/wake cycle are by now a well-established symptom of neurodegenerative diseases, and here we show that we can treat the HD symptoms by controlling the timing of food availability. The results presented in our preclinical study suggest that a TRF regimen could be a useful management tool for neurodegenerative disease patients. More generally, the present study adds to a growing body of evidence that improvements in “circadian hygiene” through attention to regularity in environmental signaling, including timed feeding, leads to improvements in health outcomes for a wide range of human diseases including neurodegenerative disorders.

## Synthesis

Reviewing Editor: Rae Silver, Columbia University Decisions are customarily a result of the Reviewing Editor and the peer reviewers coming together and discussing their recommendations until a consensus is reached. When revisions are invited, a fact-based synthesis statement explaining their decision and outlining what is needed to prepare a revision will be listed below. Note: If this manuscript was transferred from JNeurosci and a decision was made to accept the manuscript without peer review, a brief statement to this effect will instead be what is listed below.

This is a very well done study and a clear write-up of the results. The comments of the previously assigned reviewers are laudatory. One reviewer appears to have misread an essential part of the study- the use of controls. This has been noted in the rebuttal letter. All other comments were minor and have been addressed in the revised manuscript.
